# Editorial: Neurorehabilitation in neurotrauma: treating traumatic brain and spinal cord injuries

**DOI:** 10.3389/fnhum.2024.1484962

**Published:** 2024-09-10

**Authors:** Fernando Zanela da Silva Arêas, Walter Gomes Da Silva Filho, Guilherme Peixoto Tinoco Arêas, Hang Jin Jo

**Affiliations:** ^1^Baylor Scott and White Research Institute (BSWRI), Dallas, TX, United States; ^2^Baylor Institute for Rehabilitation, Dallas, NC, United States; ^3^Neurorehabilitation and Neuromodulation Laboratory, Universidade Federal do Espirito Santo, Vitoria, Brazil; ^4^Physiology Science Laboratory, Federal University of Amazonas, Manaus, Amazonas, Brazil; ^5^Department of Rehabilitation Science, University at Buffalo, Buffalo, NY, United States

**Keywords:** rehabilitation research, neurological rehabilitation, brain injuries, traumatic brain injury, spinal cord injury

Traumatic Brain Injury (TBI) and Spinal Cord Injury (SCI) represent significant global public health challenges. The World Health Organization (WHO) estimates that about 69 million individuals suffer from TBI annually, and between 250,000 and 500,000 new cases of SCI occur each year (World Health Organization, 2019). These injuries have severe consequences on the lives and social reintegration of affected individuals, highlighting the need for innovative and effective interventions to promote functional recovery (Areas et al., 2019). Neurological rehabilitation, however, encounters major obstacles due to the heterogeneity of neurological injuries, which leads to variable treatment responses and hinders the development of standardized protocols. Additionally, the complexity of these disorders often requires multidisciplinary approaches that are challenging to coordinate and resource-intensive (Ponsford et al., 2000; Turner-Stokes, 2009). Limited access to specialized care and the high costs of advanced rehabilitation technologies further exacerbate these challenges, particularly in resource-constrained settings (Chrysafides et al., 2019).

Recent advancements in rehabilitation technologies have greatly expanded treatment options. Innovations such as deep brain stimulation, virtual reality, and robotic devices are transforming traditional therapeutic approaches (Holtzheimer and Mayberg, 2011; Hidler et al., 2009; Laver et al., 2017). Deep brain stimulation has shown potential in modulating neural circuits to enhance motor and cognitive functions in TBI and SCI patients (Holtzheimer and Mayberg, 2011). Virtual reality offers immersive rehabilitation environments that enhance motor learning and patient engagement (Laver et al., 2017). Robotic devices facilitate repetitive task practice and precise movements, which are crucial for neuroplasticity and functional recovery (Hidler et al., 2009). These technologies have demonstrated promising efficacy in improving motor and cognitive functions, significantly enhancing quality of life of patients (Kleim and Jones, 2008; Alvareza et al., 2018; Hillyard and Näätänen, 2018; Edgerton et al., 2019). The ongoing development of these technologies, along with personalized rehabilitation approaches, continues to advance the field and offer innovative support for improved outcomes. In addition to these advancements, early rehabilitation efficacy is well-supported by evidence, but obstacles such as the lack of ideal predictive models and disparities in access to technologies persist, hindering progress (Kreuter and Højlund, 2020). Recent research has significantly contributed to the better understanding of these conditions.

In their scientific study, Sudhakar et al. examined psychiatric and medical comorbidities associated with mild TBI using data from the national TBI Model Systems (TBIMS) database. Their findings revealed a high prevalence of psychiatric comorbidities, including anxiety, depression, and post-traumatic stress disorder (PTSD), alongside chronic pain and cardiovascular comorbidities among survivors of mild TBI. Additionally, Xu et al. conducted a network meta-analysis to evaluate the efficacy of five Chinese medicine monomers in functional recovery in animal models of SCI. Their review of 59 studies indicated that all monomers exhibited positive effects, with tanshinone IIA (TIIA) demonstrating efficacy in early recovery and resveratrol (RSV) in later stages. The study calls for further research to enhance the standardization and clinical application of these findings. another important study developed by Castellani et al. performed a multicenter retrospective study to assess the incidence of healthcare-associated infections (HAIs) and multidrug-resistant HAIs in patients with severe acquired brain injury (sABI). Their research involving 134 participants revealed significantly higher rates of HAIs and multidrug-resistant HAIs in semi-intensive units compared to other settings. In the field of exercise-centered neurological rehabilitation, Gorgey et al. investigated the combined effects of neuromuscular electrical stimulation-resistance training (NMES-RT) and functional electrical stimulation-cycling of the lower limbs (FES-LEC) vs. passive movement training (PMT) and FES-LEC in adults with chronic SCI. The study found a trend toward increased *V°*O_2_ peak and reduced visceral fat in the NMES-RT + FES group compared to the PMT + FES group, although FES-LEC did not significantly enhance muscle cross-sectional area. However, in his scientific research, Snowden et al. conducted a systematic review of aerobic exercise as an intervention for TBI survivors. The review highlighted the effectiveness of aerobic exercise, particularly for adolescents and adults, and identified the need for additional studies focused on children and the elderly to adapt treatment guidelines for these specific populations.

An innovative study within a current context of global relevance carried out by Keleman et al. assessed the impact of the COVID-19 pandemic on early rehabilitation outcomes for TBI patients in Bosnia and Herzegovina. Analysis of data from 174 patients in 2021 indicated that the pandemic did not negatively affect clinical outcomes. Early rehabilitation remained effective, resulting in significant improvements in Glasgow Coma Scale, Barthel Index, and Functional Independence Measure scores. A clinical study performed by Zhi et al. at West China Hospital of Sichuan University was performed aiming to evaluate the efficacy and safety of acupuncture as a complementary therapy for Prolonged Disorders of Consciousness (pDOC). With 110 participants, the study aims to provide robust evidence regarding the efficacy and safety of acupuncture for pDOC, potentially informing clinical practices and future research. Eilfort et al. investigated the role of neuroplasticity in the reticulospinal (RS) and corticospinal (CS) systems in functional recovery following unilateral spinal cord injury, focusing on a patient with Brown-Séquard-plus Syndrome. Using the StartReact paradigm and transcranial magnetic stimulation (TMS), they observed a significant increase in ipsilateral RS activation in the biceps brachii, suggesting that elbow flexion recovery is primarily driven by the RS system. Eliason et al. evaluated the effectiveness of non-invasive brain stimulation (NIBS), including TMS and tDCS, both as standalone interventions and in combination with neurorehabilitation therapies. The review of 22 studies involving 657 participants revealed that while two studies found NIBS ineffective, the majority reported improvements in neuroplasticity and ABI-related symptoms.

These studies underscore the critical importance and substantial impact of neurorehabilitation research in understanding the effects of traumatic events on mortality in young individuals, years of life lost, and high incidence of disability. The breadth of interdisciplinary research in neuroscience highlighted in this editorial reflects the commitment of numerous researchers to developing effective interventions for global communities. Collectively, these studies emphasize the necessity of addressing neurological disorders as a significant public health issue, requiring targeted attention from researchers and policymakers. It is crucial that, in addition to the progress already made, ongoing research continues to tackle emerging questions to better guide efforts and investments in neurotrauma research. The significant impact of these conditions particularly on young adult mortality, years of life lost, and disability rates justifies increased allocation of resources and support. Future research is essential for deepening understanding and enhancing therapeutic strategies, and only through sustained and amplified investment can we effectively confront these challenges and reduce their associated consequences.
